# Student perception about working in rural Nepal after graduation: a study among first- and second-year medical students

**DOI:** 10.1186/1478-4491-10-27

**Published:** 2012-08-31

**Authors:** P Ravi Shankar, Trilok P Thapa

**Affiliations:** 1 Department of Medical Education, KIST Medical College, Lalitpur, Nepal; 2 KIST Medical College, PO Box 14142, Kathmandu, Nepal

**Keywords:** Curriculum changes, Developing countries, Financing, Medical students, Nepal, Rural service, Scholarship, Self-financing

## Abstract

**Background:**

The Federal Democratic Republic of Nepal is a developing country in South Asia with a population of 29.8 million. In September 2011, there were 18 medical schools with 14 being in the private sector. KIST Medical College is a private school in Lalitpur district. The present study was conducted to obtain information on student perceptions about working in rural Nepal after graduation.

**Methods:**

The study was conducted among first- and second-year undergraduate medical students using a semi-structured questionnaire developed by the authors using inputs from the literature and their experiences of teaching medical students. Year of study, gender, method of financing of medical education, place of family residence and occupation of parents were noted. Participant responses were analysed, grouped together and the number of respondents stating a particular response was noted.

**Results:**

Of the 200 students, 185 (92.5%) participated with 95 being from the first year and 90 from the second. Most students were self-financing and from urban areas. Regarding the question of working in rural Nepal after graduation, 134 (72.4%) said they will work after their undergraduate course. Students preferred to work in the government or nongovernmental sector. Student felt doctors are reluctant to serve in rural Nepal due to inadequate facilities, low salary, less security, problems with their professional development, less equipment in health centres, decreased contact with family and difficulties in communicating with an illiterate, rural population. About 43% of respondents felt medical education does not adequately prepare them for rural service. Repeated rural exposure, postings in rural hospitals and health centres, and training students to diagnose and treat illness with less technology were suggested. The median monthly salary expected was 60 000 Nepalese rupees (US$ 820) and was significantly higher among first-year students.

**Conclusions:**

The majority of respondents were in favour of working in rural Nepal after graduation. They wanted facilities in rural areas and health centres to be improved. Changes in the education system were suggested. Providing relatively better facilities for rural doctors compared with urban doctors and reorienting medical education for producing doctors for rural Nepal can be considered. Further studies are required in other private medical schools.

## Background

The Federal Democratic Republic of Nepal is a developing country in South Asia situated between China and India. The population of Nepal in the year 2010 was 29.8 million [1]. There were 18 medical schools in the country at the beginning of September 2011 [2] and 14 of these were in the private sector. Nepal is predominantly an agricultural country and, in 2009, 24.1 million people (about 90% of the population) stayed in rural areas [3]. In Nepal, doctors migrate to developed nations or prefer to work in urban areas of the country. On calculating the number of doctors per 10 000 population in each district according to 2001 census population data, 5 districts had fewer than 1.5 doctors, 32 had between 1.5 and 3 doctors, 31 had between 3 and 5 doctors, and 7 had more than 5 doctors [4]. The situation may have changed as many new medical schools have been established in the decade following 2001.

In Nepal, private medical schools run by foreign groups have to provide 20% of their total seats on full tuition fee scholarship for students selected through an entrance examination conducted by the Ministry of Education [5]. Schools run by Nepalese groups have to provide 10% of seats to scholarship students. These students have to serve two years in rural areas after graduation and are becoming an important source of support to Nepal’s health system [5].

KIST Medical College (KISTMC) is a private medical school in Lalitpur district of the Kathmandu valley committed to excellence in holistic healthcare, education and research. The college is affiliated to the Institute of Medicine, Tribhuvan University for the undergraduate medical (MBBS) course. The six basic science subjects of anatomy, physiology, biochemistry, pathology, pharmacology and microbiology are taught in an integrated, organ system-based manner during the first two years. Community medicine occupies an important place in the curriculum and students spend time in semi-rural communities (community diagnosis programme) after finishing the academic activities of the first year of the course. The community diagnosis field posting is held towards the end of the first year after the organ systems have been covered. The residential field posting has the objectives of enabling students to communicate with the rural and semi-rural population about different health problems observed in the community, and to apply different data collection techniques to study, analyse and prioritize these problems [6]. Students also learn to identify community resources to solve the problems, and plan and implement a micro health project with community participation to address the priority health needs. At KISTMC, students spend two weeks in semi-rural communities at the outskirts of the Kathmandu valley and develop close links with the communities where they are posted. They are assessed on their residential field posting during their community medicine practical examination at the end of the first year.

In the United Kingdom of Great Britain and Northern Ireland, rural clinical placements have been developed to train medical students for practice in rural areas. Student perceptions about rural placements were negative, citing reasons like narrow range of patient contact and learning opportunities and rural life being considered unattractive [7]. Many countries have used financial incentive programmes to address health worker shortages in rural and underserved areas. A recent systematic review concluded that these programmes have placed substantial numbers of health workers in rural areas and programme participants are more likely to work in rural areas [8]. However, the review concluded that available evidence to date does not allow the inference that financial incentive programmes have increased health worker supply to underserved and rural areas. A recent review of the literature concluded that there are predictable factors which influence recruitment and retention of primary care physicians in rural areas [9]. These factors are selecting the right students, and giving them proper training, including the experience required to succeed in primary care in rural areas.

In Canada, the majority of medical schools have either mandatory or elective rural medicine placement or learning experiences during undergraduate medical education, as well as rural family medicine streams. They provide traineeships to enhance clinical competencies in rural medicine as well as continuing medical education outreach programmes, including the use of telehealth or distance learning technologies [10]. A Canadian study has examined the role of medical education in recruiting and retaining rural doctors [11]. The authors state that rural student recruitment, admissions policies, a rural-oriented medical curriculum, rural practice learning experiences, faculty values and attitudes, and advanced procedural skills training increase the likelihood of medical students entering rural primary care practice. A study conducted among undergraduate medicine, nursing and pharmacy students has shown that professional and peer support, work conditions and variety of work were important conditions governing career choice [12].

The perception of medical students about working in rural Nepal after graduation is an important area of study. The increasing numbers of private medical schools and self-financing students means that these students will form a major proportion of the medical workforce. Hence, their perceptions about working in rural Nepal become increasingly important. This study was conducted among first- and second-year undergraduate students with the following objectives:

 · to obtain basic demographic information of the student respondents;

 · to understand respondent perceptions about why doctors are reluctant to work in rural Nepal and important conditions they feel should be fulfilled to work in rural areas;

 · to study problems of working in rural Nepal and obtain possible solutions;

 · to obtain information on the minimum monthly salary that respondents expect to receive to work in rural Nepal.

## Methods

The study was conducted during August and September 2011 among first- and second-year students at KISTMC. The questionnaire was developed by the authors using inputs from the literature and their experiences of teaching medical students. Information from a recently concluded study on the possible implications of the increasing number of female medical students in Nepal was also used. The questionnaire was tested for readability and ease of comprehension among four third-year students.

The study was approved by the Institutional Review Board of KISTMC. The aims and objectives of the study were explained to students and they were invited to participate. Written informed consent was obtained from all participants. Only students willing to participate in the study were chosen. The Institutional Review Board insisted that participation in the study be voluntary and those not interested should not be coerced to participate. Year of study, gender, method of financing of medical education, place of family residence and occupation of parents were noted. Student perceptions about working in rural Nepal after graduation were studied using a semi-structured questionnaire shown in the Additional file 1.

Participant responses were analysed, grouped together and the number of respondents stating a particular response (frequency of response) was noted. Among the areas studied were whether students planned to work in rural Nepal after graduation, what they would consider as important conditions to be fulfilled before they consider working in rural Nepal, reasons why doctors are reluctant to work in rural areas, how the government can encourage doctors to work in rural Nepal, possible problems in dealing with and providing healthcare to the rural population, the problem of return of investment on the high tuition fees paid and the minimum monthly expected salary required to work in rural Nepal.

The demographic information, willingness to work in rural Nepal and the minimum monthly salary were entered into SPSS version 17 for Windows. The normality of distribution of salary among respondents was checked using a one sample Kolmogorov-Smirnov test. The distribution was not normal and the median salary was compared among first- and second-year students, scholarship and self-financing students, male and female students and urban and rural students using appropriate non-parametric tests. A *P*-value less than 0.05 was taken as statistically significant.

The free text responses to questions in the questionnaire like, ‘Three important conditions to be fulfilled before you can consider working in a rural area’ were noted verbatim by the authors. These were then grouped into categories of responses according to similarities among the responses. The frequency of these response categories was then noted.

## Results

Out of 200 first- and second-year students, 185 (92.5%) participated. If third-year students are also included, then 67.3% of the enrolled student population at the time of the study (275 students) participated in the present study. The third-year students were not included in the study due to logistic difficulties. The participating students comprised 95 first-year students and 90 second-year students. Table 1 shows the demographic characteristics of student respondents. Many respondents did not mention the occupation of their mothers. Among those mentioned, many were housewives while others were teachers. Four were business women.

**Table 1:** Demographic characteristics of respondents

**Characteristic**		**Number (%)n =185**
Year of study	First	95 (51.4)
	Second	90 (48.6)
Gender^a^	Male	94 (50.8)
	Female	90 (48.6)
Financing^a^	Scholarship	20 (10.8)
	Self-financing	164 (88.6)
Family residence^a^	Urban	152 (82.2)
	Rural	21 (11.4)
Occupation of father	Business	53 (28.6)
	Government service	49 (26.5)
	Paramedic/doctor	12 (6.5)

^a^The responses may not add up to 185 as some respondents did not answer the question.

Only 21 students (11.3%) had lived in a rural area before joining the college. Twenty-eight students (15.1%) had relatives working in government service in rural areas. Regarding the question of working in rural Nepal after graduation, 134 (72.4%) said they would work there, while 3 said they would after their MD (postgraduate degree), 31 (16.8%) said they will not work in rural areas, while 17 were undecided. Eighty-five respondents (45.9%) said they will work rurally for 1 to 2 years, 15 (8.1%) stated for 3 to 4 years, 12 (6.5%) for 4 to 5 years while 13 (7.0%) were undecided. Regarding their future place of work, 92 respondents (49.7%) preferred a semi-urban area. A respondent preferring to work in a rural area wrote, “*I am from the rural area. I know the condition and need of medical facility there. Even a small/minor illness takes lives. So, I prefer to work there. Moreover I don’t like crowded, polluted city area*”.

Table 2 shows the preferences of respondents for working in government, nongovernmental organizations (NGOs) or the private sector and reasons for the same. Problems of the government sector and political interference were cited as reasons for preferring NGOs or the private sector. Among important conditions to be fulfilled for working in rural areas were a good, competitive salary (100 respondents, 54%); improvement in transportation and communication facilities (74 respondents, 40%); adequate security (70 respondents, 37.8%); good working conditions (50 respondents, 27%); provision of family accommodation (43 respondents, 23.2%); basic living facilities (40 respondents, 21.6%); availability of medicines in the health facility (21 respondents, 11.4%); and adequate health personnel in the health facility (19 respondents,10.3%). Among other conditions mentioned were lack of political interference, facilities and scholarships for further study and changes in medical education to prepare doctors for rural service.

**Table 2:** Preferred sector of working with reasons

**Sector**	**Number (%)**	**Reasons (number of respondents)**
Government	76 (41.1)	Stable job (18)
		Helping the nation (16)
		More patients (15)
Nongovernmental organization	70 (37.8)	More money (29)
		Better diagnostic and treatment facilities (19)
		Contributes more to people’s welfare (14)
		More professional approach (11)
Private	27 (14.6)	Better working conditions (16)
		More salary (10)

Seventy-six respondents (41.1%) stated they will consider working in rural Nepal immediately after graduation while 89 (48.1%) said they will consider it later in their career. Table 3 shows common reasons mentioned for why doctors are reluctant to serve in rural areas. Among other reasons cited were difficult life, lack of educational facilities for children, desire for luxury among doctors, adjustment difficulties, too much pressure on one doctor and lack of technology. Among the various measures which the government could implement to encourage doctors to opt for rural service were improving facilities (102 respondents, 55.1%), increasing the salary (90 respondents, 48.6%), providing improved security to doctors in rural areas (40 respondents, 21.6%), and scholarships for higher education (29 respondents, 15.7%). Among other measures mentioned were to also send experienced doctors, a rotation system of working, group practice and a stable political climate. Rural development, less political interference and a regular supply of medicines were also mentioned. A respondent wrote, “*Government should manage at least two doctors at one centre so that the doctors can work six months in rural area and six months in urban. While working in urban area doctor will be able to learn more from his/her colleague*”.

**Table 3:** Common reasons why first- and second-year medical students in the study are reluctant to serve in rural areas

**Reason**	**Number (%)**
Lack of adequate facilities	114 (61.6)
Inadequate salary	86 (46.5)
Less security	58 (31.4)
Problems with higher education	37 (20.0)
Less medical equipment in health centres	29 (15.7)
Less contact with family	27 (14.6)
Illiteracy of rural population	15 (8.1)

Seventy-nine respondents (42.7%) felt that their medical education did not adequately prepare them for rural service. Ninety-six respondents felt the education was adequate. Among the suggestions to refocus education for rural practice were repeated rural exposure with students spending adequate time in rural areas (39 respondents, 21.1%), training students to diagnose and manage diseases with less technological aids (26 respondents, 14.1%), more focus on diseases of rural Nepal (11 respondents, 5.9%) and conducting motivational programmes for medical students (11 respondents, 5.9%). Other suggestions were having faculty who have served in rural areas, improving communication skills of students, making students more self-reliant and having more community-based teaching. A respondent stated, “*It is very difficult to get all the equipments in rural area. So doctor should be able to diagnose disease on the basis of history taking and physical examination. Today’s teaching is dependent on lab investigations and technology for diagnosis*”.

Ninety-eight students (53.0%) felt they were aware of the problems of life in rural Nepal while 82 (44.3%) felt they were not fully aware. Among methods suggested to create awareness were regular trips to rural areas, rural hospital service during training, an orientation programme for students, using documentaries and videos, and conducting community diagnosis programmes in rural areas. Among the problems foreseen by students were communicating with the patient (63 respondents, 34.1%), problems due to illiteracy of rural population (48 respondents, 25.9%), problems due to different languages (42 respondents, 22.7%), preference of the rural population for traditional healers (31 respondents, 16.8%), problems due to differences in culture (27 respondents, 14.6%), rural population being less willing to accept modern, western medicine (23 respondents, 12.4%) and superstitious beliefs of the rural population (19 respondents, 10.3%). Among other problems suggested were that patients come very late for treatment, patients may be more aggressive, doctors are not adequately trained for situations they will encounter, and high expectations of patients. A male, self-financing respondent wrote, “*We will be dealing with a population where almost all daily activities are according to their social values and norms and we might be facing problems of false beliefs and others associated with the norms*”.

Table 4 shows suggested solutions for problems of working in a rural area and dealing with the rural population. Other suggestions included rural development, investing more on the health sector, making available senior doctors for consultation, and providing counselling and support facilities for doctors. A respondent wrote, “*Training to the doctors before sending them to rural areas about the customs, traditions and language. It can also be done that doctors who are originally from that place can be given preference to work in that area*”. Sixty students (32.4%) were aware of initiatives to address the shortage of doctors in rural Nepal. Thirty students (16.2%) stated that they were aware of the two-year compulsory rural service for scholarship students. Other initiatives mentioned were incentives to government doctors working in rural areas, private groups paying a good salary for doctors serving in rural areas, scholarships for postgraduation, and starting of the Patan Academy of Health Sciences (PAHS). Only 21 students (11.4%) were aware of similar initiatives in other countries. A large number of students (153, 82.7%) were of the opinion that a poor return on investment may be a major factor preventing doctors from serving in rural areas. Forty-three students (23.2%) were of the opinion that, as a heavy price had been paid for education, students may feel they have to recover the same. Other reasons mentioned were less money in rural areas, the greed and need for money being a part of human nature, becoming a doctor is hard work, and doctors are used to a good lifestyle. A female self-financing respondent stated, “*Yes, because the self-financing students who become doctors hesitate to work in rural areas because they need to earn the sum that they paid while studying. In rural areas there might be less outcome leading to preventing doctors from moving there*”. Another stated, “*We are not running a charity with the hard-earned money of our parents*”.

**Table 4:** Suggested solutions by the student respondents to problems of working in a rural area and dealing with the rural population

**Solution**^**a**^	**Number (%)**
Creating awareness among people about modern medicine	72 (38.9)
Training medical professionals to meet challenges of rural health	30 (16.2)
Improving general education in rural areas	20 (10.8)
Providing health education to the rural population	18 (9.7)
Arranging orientation program for doctors before posting in rural areas	12 (6.5)
Decreasing the gap between rural people and doctors	10 (5.4)
Providing medicines at cost price and free medical consultation	10 (5.4)

^a^These are suggested solutions by first- and second-year medical students. The relevance of these suggested solutions have been partly discussed in the Discussion. Many of these may be ‘generic’ solutions and more studies may be needed to obtain feasible solutions which can be implemented in practice.

Regarding the minimum monthly salary, most students expected between 50 000 Nepalese rupees (NRs) (US$ 690) and NR 100 000 (US$ 1380). The median expected salary was NR 60 000 (US$ 820). The median expected salary was significantly higher among first-year students. Their expected median salary was NR 75 000 (US$ 1030) compared with 50 000 (US$ 690) among second-year students (*P* = 0.003). A large majority of students (168, 90.8%) wanted a dedicated rural health service to be established in the country. They believed that the service should ensure good working conditions in rural areas, have a separate budget, and could use the model of public-private partnership among others. Among other comments, respondents wanted moral values to be incorporated in the curriculum. A respondent stated, “*Rural areas are also a part of Nepal!! Nepal is developed only after development in rural sector*”. A female respondent wrote, “*I would like to work in a rural area as I myself come from a remote village. But I am not going to compromise with my future*”. Another stated, “*This will be a great initiative if the objectives of this research are accomplished and the necessary steps taken*”.

## Discussion

Most respondents were from an urban area and had not lived in a rural area. In Nepal, the majority of students are from the Kathmandu valley and prefer to study if possible in colleges near their place of residence. This could be one of the reasons for a large number of students being from urban areas. Most schools are located in urban areas and admit predominantly self-financing students (80% of students in foreign-owned schools and 90% in Nepalese-owned ones as stated previously).

Nearly 72% of respondents stated they will work in rural Nepal for a period of time. Many preferred a semi-urban area as their work location. Most preferred working for the government or the NGO sector. A good competitive salary, good infrastructure, proper working conditions and adequate security were prerequisites. Scholarships for higher education could also motivate doctors to work in rural areas. Many felt that their education did not adequately prepare them for rural practice and suggested modifications. High tuition fees and the question of return on investment were suggested to be factors preventing doctors from working in rural areas. The number of women medical students is increasing in Nepal, with implications for medical education and health-care delivery [13].

A shortage of doctors in rural areas is a major problem not just in Nepal but internationally. Strategies employed in the United States of America include employing international medical graduates as primary care physicians in rural areas [14], offering financial incentives in the form of loan repayment and scholarships for students willing to serve in rural areas [15] and developing medical education programmes that offer special curricula preparing doctors for rural practice, among others. In our study, over 72% of respondents indicated they will work for a period of time in rural areas. In an Australian study, 38.5% of first-year and 56.3% of final-year students indicated a preference for rural life and practice and over 90% of respondents indicated they will spend at least some time in rural practice [12]. In a second study from Australia, the themes identified as predictive of rural service were rural origins of the students, presence of rural origin people on the selection panels and rural experience during the medical course [16]. In Nepal, students are selected on the basis of their performance in an entrance examination conducted by the Ministry of Education or respective universities. Foreign students are selected based on an interview. The financial status of parents and ability to pay tuition fees are important factors considered during selection. International graduates migrating to work in Nepal may not be an option because of the poor living and work conditions offered. Foreign doctors may volunteer for a limited period of time due to the unique topography of the country, offering opportunities in high altitude medicine and research, and other reasons. Financial incentives in the form of loan repayment have not been offered in Nepal. Full tuition fee scholarship is offered for selected students in private schools with the obligation to work in rural health facilities for two years after graduation.

The creation of a rural pipeline starting with structured contact between rural secondary schools and the medical profession, rural student selection into medical programmes, rural exposure during training and measures to retain the workforce in rural areas have been tried in developed nations. A period of rural residence of unspecified length has been stated to be the strongest predictor of rural service after graduation [17,18]. Many countries have implemented quotas for rural area students, adjusting selection scores according to the origin of candidates. In Nepal, seats are reserved for candidates according to caste and ethnicity for disadvantaged sections. Recently, PAHS has been started to create doctors for rural Nepal [19]. PAHS admits 60 students to the MBBS programme and gives preference to applicants from rural Nepal, to socially disadvantaged groups and to health assistants who have served at least two years in a rural area. PAHS also has a scheme where rural communities or hospitals can sponsor students to study an MBBS with the provision that they will return to serve the community for a specified time period after graduation. Students spend time with rural families and learn to adapt to life in rural Nepal.

Australian students stated poor clinical learning experiences during training and the perception that career opportunities may be limited were significant impediments to rural service. Students are not equipped with skills necessary to work in primary care settings in rural areas. External factors like living with a partner who was not committed or was not able to live in a rural area, personal preference for an urban lifestyle and isolation from family and friends were also stated to be responsible factors [16]. The factors mentioned in this Australian study were similar to those in our study. A study in the Kingdom of Thailand showed that family and community commitments, a sense of belonging and high social status were reasons behind health-care professionals choosing to work in their province of origin [20]. In our institution, the number of students from rural areas was low. Many rural families have recently migrated to urban areas. A similar situation may be seen in other medical schools. Hence family and community commitments may not play an important role in motivating students to work in rural areas. Financial remuneration, promotions and professional development opportunities were also perceived as important in the study from Thailand, similar to the data in our study.

In a study in the Socialist Republic of Vietnam, salary levels, allowances, ability to combine work with private practice, availability of equipment, working conditions, workload, social relationships, access to training, professional development, social relationships, children’s education, living conditions in the area of posting and housing were regarded as important factors [21]. A recent article from Nepal suggests using ‘the five Cs’ to keep health workers happy and productive [22]. These are communication, continuing medical education, connection with a higher hospital, community management of hospitals, and children’s education. Communication facilities are improving and many hospitals are being managed by rural communities. Education of children and continuing medical education continue to be problems in the Nepalese setting.

The issue of curriculum modifications to prepare students for rural practice is receiving attention all over the world. At the University of Illinois in the United States of America, students can opt for a rural medical education programme [23]. There are special rural-focused topics during the first three years of undergraduate medical education and a required fourth year rural preceptorship where students work with primary care physicians and conduct community-oriented primary care projects. Decentralized medical schools located in rural areas which encourage admission of rural students, have a rural-oriented curriculum and offer repeated rural practice experience to their students are most successful at graduating physicians who choose rural practice as a career [24]. In Nepal, while some sections of a medical school are located in rural areas, none can be considered as a rural medical school. Clinical training mainly takes place in teaching hospitals attached to the college.

A study in Australia had divided students into ‘true believers’, those from a rural background who chose a rural career path; ‘convertibles’, students from an urban background choosing a rural career path; ‘frustrated’, students from a rural background choosing an urban career path; and ‘metro docs’, those from an urban background who choose an urban career path [25]. The true believers were predominantly female married to a rural partner and who had spent long periods of time in rural areas. The convertibles were mostly urban females fond of rural practice and disliking city living. In Nepal, the majority of students are from urban areas and the conveniences available in Australia may be lacking in rural Nepal.

In Nepal, rural population often prefers traditional healers. A possible reason could be that these healers are from the rural community itself, and are always available to provide care to the rural population. Traditional healers have been used to deliver healthcare in Nepal and other developing countries. In Nepal, they have been used to deliver healthcare near Kavrepalanchowk district near Kathmandu [26] and to deliver primary eye care [27]. In our study, there seemed to be a lack of trust between traditional healers and allopathic doctors. This has been noted in the literature [28] and greater understanding and cooperation between doctors and traditional healers may be required for more efficient, rural health-care delivery.

In India, many initiatives were carried out under the National Rural Health Mission to retain health workers in rural areas [29]. Among these initiatives were an increase in sanctioned posts in public health facilities, incentives, workforce management policies, locality specific recruitment and creation of a specific cadre for public sector employment. A recent survey in Nepal showed that students who indicated likelihood for rural practice were more likely to be male, to have been born in a village, to have studied in a government secondary school and to have received a government scholarship [30]. The majority of students agreed with a policy necessitating all students to practice for a period of time in rural areas, and the authors of that study suggested that rural postgraduate courses weighted towards requirements of rural Nepal be started [30]. A recent article suggested a range of strategies used elsewhere could be of benefit in Nepal. These included community-oriented medical education, reimbursement of tuition fees, assistance with relocation, provision of academic and professional advancement opportunities and government investment in improving working conditions in rural areas [4].

The issue of return of investment and tuition fees charged by private medical schools may require serious consideration. In the year 2010, KISTMC raised tuition fees by around 30% and the first-year students were more reluctant to serve in rural areas and expected a significantly higher median monthly salary. At present, most medical schools charge tuition fees of around NR 3.5 million (US$ 45 000), a significant amount in a poor country. Recently the government has recommended a ceiling of NR 2.5 million for tuition fees. The impact of this is awaited. This has to be viewed in the light of the fact that the government does not support private medical schools and the major source of income is student fees.

The strength of our study is the high response rate and free and frank response of participants. The study had limitations. It was carried out only in a single medical school in Nepal. The students were in the first two years of the MBBS course only; third-year students were not included. The literature shows there may be a shift in career preferences of medical students as they progress through medical school, thus the omission of third-year students is a limitation. This issue can be explored in the future once we have students in all years of the undergraduate medical course. The study was carried out among students in an urban medical school with predominantly self-financing medical students. However, this is fairly representative of medical students because, as mentioned in the discussion, most medical schools in Nepal are in urban areas and admit predominantly self-financing students. Student response was obtained using a questionnaire and was not compared with information from other sources. The sample size was low compared with some other studies from the literature. There is a certain amount of repetition of responses to different questions. A similar study should be carried out among students in other medical schools and among all the years of study.

## Conclusions

The majority of medical students surveyed were in favour of working in rural Nepal after graduation. Respondents recommended a good, competitive salary, adequate security and proper working conditions as prerequisites. Suggestions included increasing the salary, improving facilities in the workplace and in rural areas, adequate security for doctors and facilities for higher education. Group practice or more than one doctor in a health facility could be considered. Changes in the education system were suggested, with students repeatedly spending time in rural areas, trained to function with less technology, and motivated for rural service. Students were not comfortable with rural populations and so familiarization sessions and training on communicating better with villagers may be required. High tuition fees and return on investment may influence choice of work for the doctor. In contrast to a previous study, scholarship students were not significantly more likely to serve in rural areas and there was no difference in expected monthly salary. Improving facilities for doctors in rural areas, providing relatively better facilities compared with urban doctors and reorienting medical education for producing doctors for rural Nepal can be considered.

The specificity of these conclusions can be improved by using these conclusions as a base to help medical students in this medical school and in other medical schools in the country reflect on concrete measures which could be taken to increase the number of doctors in rural Nepal.

## Competing interests

The authors declared that they have no competing interests.

## Authors’ contributions

PRS was involved in the design and conduct of the study, analysis of data, review of the literature and writing the manuscript. TPT helped in the design and conduct of the study, the review of the literature and helped in writing the manuscript. Both authors have read and approved the final manuscript.

## Supplementary Material

Additional file 1 **Appendix:** Questionnaire used in the study. Click here for file
