# Supplementary material for: Student perception about working in rural Nepal after graduation: a study among first- and second-year medical students
Source: Hum Resour Health. 2012 Aug 31;10:27. doi: 10.1186/1478-4491-10-27 (PMC3464161; doi:10.1186/1478-4491-10-27)
Supplement: Additional file 1 — Appendix: Questionnaire used in the study. [file 1478-4491-10-27-S1.doc]

**Appendix:** Questionnaire used in the study

**Student perception about working in rural Nepal after graduation**

Year of study: Gender: Male/Female Scholarship/Self-financing Family residence: Urban/Rural Occupation of parents:

Were you living in a rural area before joining KIST Medical College?

Any relatives in rural government service: Yes/No If yes, please provide details:

Do you plan to work in rural Nepal after graduation?

If yes, then for how many years? What will be your preferred place of work? (Urban/rural/semiurban)

Will you prefer to work in the government/NGO/Private sector in rural areas?

What are the reasons for your preference?

What are THREE important conditions to be fulfilled before you can consider working in a rural area?

Will you consider working in rural Nepal immediately after graduation or later in your career?

According to you why are doctors reluctant to work in rural Nepal (three most important reasons)?

According to you how can the government encourage doctors to serve in rural Nepal?

Does your medical training adequately prepare you for a career in rural practice? Yes/No

If no, then can you make THREE important suggestions to refocus medical education for practice in rural Nepal.

Are medical students aware of problems of life in rural areas?

If no, then how can this awareness be created?

What problems in dealing with the local population do you foresee if working in a rural area after graduation?

How can these problems be addressed?

Are you aware of any initiatives in Nepal to address shortage of doctors in rural areas?

Are you aware of similar initiatives in other countries?

Do you think poor return on investment is a major factor preventing doctors from working in rural areas? Why?

What should be the minimum monthly salary which according to you will be attractive for working in rural Nepal after graduation?

Do you think a dedicated Nepal rural health service of doctors serving in rural Nepal should be created?

How should the government go ahead with creating the rural health service?

Any other comments:

Thank you for taking the time to fill the questionnaire!
